# Effects of inbreeding on reproductive success in endangered North Atlantic right whales

**DOI:** 10.1098/rsos.240490

**Published:** 2024-07-31

**Authors:** Carla A. Crossman, Philip K. Hamilton, Moira W. Brown, Lisa A. Conger, R. Clay George, Katharine A. Jackson, Sonya N. Radvan, Timothy R. Frasier

**Affiliations:** ^1^ Biology Department, Saint Mary's University, Halifax, Nova Scotia, Canada B3H 3C3; ^2^ Anderson Cabot Center for Ocean Life, New England Aquarium, Central Wharf, Boston, Massachusetts, USA; ^3^ Canadian Whale Institute, Welshpool, New Brunswick, Canada; ^4^ NOAA Fisheries, Northeast Fisheries Science Center, Woods Hole, MA, USA; ^5^ Georgia Department of Natural Resources, Wildlife Conservation Section, Brunswick, GA, USA; ^5^ Florida Fish and Wildlife Conservation Commission, Fish and Wildlife Research Institute, Saint Petersburg, FL, USA

**Keywords:** ddRAD sequencing, North Atlantic right whale, inbreeding depression, inbreeding, conservation, heterozygosity-fitness correlations

## Abstract

Only approximately 356 North Atlantic right whales (*Eubalaena glacialis*) remain. With extremely low levels of genetic diversity, limited options for mates, and variation in reproductive success across females, there is concern regarding the potential for genetic limitations of population growth from inbreeding depression. In this study, we quantified reproductive success of female North Atlantic right whales with a modified de-lifing approach using reproductive history information collected over decades of field observations. We used double-digest restriction site-associated sequencing to sequence approximately 2% of the genome of 105 female North Atlantic right whales and combined genomic inbreeding estimates with individual fecundity values to assess evidence of inbreeding depression. Inbreeding depression could not explain the variance in reproductive success of females, however we present evidence that inbreeding depression may be affecting the viability of inbred fetuses—potentially lowering the reproductive success of the species as a whole. Combined, these results allay some concerns that genetic factors are impacting species survival as genetic diversity is being retained through selection against inbred fetuses. While still far fewer calves are being born each year than expected, the small role of genetics underlying variance in female fecundity suggests that variance may be explained by external factors that can potentially be mitigated through protection measures designed to reduce serious injury and mortality from human activities.

## 1. Introduction

Globally, the largest threats to biodiversity are anthropogenic in nature, but as population sizes dwindle, further risks associated with small populations remain, and can be much harder to mitigate. Genetic diversity erodes faster in small populations due to genetic drift [1], and limited options for mates leads to mating between related individuals, which can compromise the health and fitness of offspring known as inbreeding depression [2–5]. Spielman *et al*. [6] found that genetic factors impact species long before they go extinct but genetic effects are inherently difficult to manage in most species. Introducing individuals from other populations to increase genetic diversity, known as genetic rescue, has been effective in some cases such as an isolated population of grey wolves (*Canis lupus*) that benefitted from the arrival of an immigrant [7] or in an endangered population of adders (*Vipera berus*) where new males were introduced [8], which both resulted in rapid population growth. However, when an entire species is endangered, or when the size or general biology of a species limits the practical application of genetic interventions, little can be done to mitigate the impacts of genetic diversity directly. In these cases, recognizing the impacts reduced genetic diversity and inbreeding are having on the species is still essential to conservation management to understand genetic limitations on population recovery and/or resiliency.

Inbreeding depression is being increasingly reported in wildlife populations with effects on many different measures of fitness such as parasite infection [9], maternal effects on offspring growth [10], lifetime breeding success [11,12], and survival [10,12,13], but barriers to its detection still exist. Inbreeding depression is more likely to affect life-history traits than morphological traits [14,15], however there can be practical limitations to collecting these data in wildlife populations. For example, lifetime reproductive success may not be available for long-lived species, and limited resources for field work may result in missed detection of offspring. Furthermore, the true strength of inbreeding depression may be masked if the survival of highly inbred individuals is lower, leading to them being under-sampled. This was demonstrated in a study on harbour seals (*Phoca vitulina*) where inbreeding was associated with lungworm burden in young animals but not in older individuals presumably because more inbred animals succumbed to infections early in life and at higher rates than non-inbred individuals [16]; therefore assessing inbreeding depression in harbour seals by studying only adults would have failed to detect evidence of inbreeding depression entirely. Thankfully, methods to quantify inbreeding in individuals have improved in recent years. Genomic estimates of inbreeding are now easily accessible and far outperform in their ability to capture realized inbreeding across many markers, and this has also opened the doors for the number of ways we can measure or quantify an individual's inbreeding coefficient. Inbreeding can be assessed with measures of heterozygosity such as standardized multi-locus heterozygosity (sMLH; [17]) or homozygosity by loci (HL) which was developed specifically to assess heterozygosity fitness correlations [18]. Inbreeding ancestry can also be measured by looking at runs of homozygosity (ROH) or stretches of the genome that are homozygous by descent (HBD). Recent inbreeding leads to longer ROH, whereas the ROH from older inbreeding events tend to break up into shorter segments by recombination over time [19]. Evaluating inbreeding depression with different inbreeding coefficients may help disentangle its drivers and better understand how inbreeding is affecting, and will continue to affect, the species [20].

North Atlantic right whales (*Eubalaena glacialis*) are an endangered species of baleen whale that inhabit the near coastal waters of eastern North America. An estimated 356 individuals remain and the population has been declining for the past decade due to a combination of high mortality and low reproduction [21]. Vessel strikes and entanglement in commercial fishing gear are the leading causes of mortality [22–24]. In addition, the sublethal effects of changes in food resources linked to climate change [25] and of anthropogenic activities [26] are having widespread impacts. Population growth in the species is facing even more challenges with a heavily male-biased sex-ratio [27] and females taking longer to transition from immature to breeding adults [28]. Furthermore, females are not reproducing as frequently as expected with calving success only 27% of what would be expected [29] and 36 reproductive age females have not produced a known calf [30]. Based on the congeneric southern right whale (*E. australis*) and on the reproductive histories of some North Atlantic right whales, females should be capable of reproducing every three years [31,32], yet few females are living up to that expectation and there is large variance in calving output with some females calving reliably every few years, while others only produced one calf and some never produced a calf [30].

Previous research has shown that North Atlantic right whales have extremely low levels of genetic diversity [33–36], have had a relatively small effective population size throughout much of their history [36], and show signs of relatively high rates of recent inbreeding [36]. These factors suggest that the poor reproductive success may be a result of inbreeding depression. North Atlantic right whales have been the focus of long-term field studies for over 40 years—with a significant increase in field effort beginning in the mid-1980s [37]. Individual whales can likely live for over 70 years [38] and are uniquely recognizable after their first year based on external markings [39]. Also, calves stay in close proximity to their mothers while they are nursing for their first year of life [40], which has allowed many females' reproductive success to be tracked through time. Combined, these decades of individual-based data provide a wealth of information that forms the basis for our understanding of this species and for testing hypotheses regarding factors impacting health, mortality, and reproduction; as well as for better understanding general aspects of biology and evolution (e.g. [41,42]).

To estimate individual reproductive fitness, a metric is needed that is not biased by observer effort or the underlying biology of the species. Comparing the age at first calving as a measure of fitness could be biased toward younger females, for which their year of birth is known (whereas older females were born before studies began and are therefore of unknown age). Lifetime reproductive output requires confidence in her first calving attempt and would underestimate the reproductive capacity of living females or females killed by anthropogenic activities who still have (or would have had) many calving years ahead of them. Estimating survivorship of calves beyond their first year would be interesting, but calves cannot be individually identified until the latter half of their first year, and therefore the fate of some calves is not known, and such analyses would have an inherent bias in detecting calves that survived their first year. Ideally, North Atlantic right whale female reproduction should follow a three year cycle where a female is pregnant for a year, suckles a calf for a year and then has a year of recovery before becoming pregnant again [31]. Quantifying the inter-calf interval for a female as the mean number of years between calves could be an indicator of fitness if it accurately represents her ability to recover from pregnancy and lactation; however, if a calf dies very young, some females reproduce next with a two year interval which can be interpreted in different ways. It could either be biologically ‘impressive’ for a female's ability to reproduce, or it could signify she produces unfit calves. Moreover, because our data are time-limited, there will always be a bias towards observing more shorter intervals than longer intervals, because there are more opportunities to observe shorter intervals. Such a metric would also ignore those females who have never given birth or have had only one calf.

Coulson *et al*. [43] proposed a measure to estimate relative fitness called the ‘de-lifing method’ that overcomes many of the limitations described above. It combines a survivorship and a fecundity component to quantify an individual's relative lifetime contribution to population growth. One of the strengths of this method is that it scales an individual's contribution based on what is happening with others in the population—for example reproducing in a year when everyone else reproduced is weighted less heavily than reproducing in a year when very few offspring are born. Likewise, surviving a year with high mortality is weighted more heavily than surviving a year in which few individuals die. The fecundity component of the de-lifing method may be well suited to measure reproductive fitness in female right whales as it allows for quantifying an individual's relative reproductive success within the context of population-wide trends and fluctuations. In right whales, our fitness measurements will still suffer from an observation bias, however by using a de-lifing approach to measure fecundity, we can overcome some of the temporal fluctuations in the environment, such as changes to food resources which we know are having an increasing effect on the species [25].

North Atlantic right whales are critically endangered [44], have a low effective population size [36] and estimated abundance [21], and few females are reaching their maximum reproductive potential [28,30]. If inbreeding is having a strong influence on reproductive success, understanding this relationship could be extremely important for understanding genetic limitations on population growth. Weaker patterns could be indicative of external confounding factors affecting reproductive performance and could suggest that inbreeding may not be a main limitation to population growth—yet. If there is no correlation between inbreeding and reproductive success, inbreeding depression may still help explain poor reproductive success in the species, just not through reduced female fecundity. We used reduced representation genome sequencing to estimate inbreeding coefficients for 105 female North Atlantic right whales and used known calving history data to investigate the effects of inbreeding on reproductive success.

## 2. Material and methods

### 2.1. Sample selection

The North Atlantic Right Whale Consortium (www.narwc.org) collaboratively maintains a database of sighting histories and life-history data, as well as a DNA/tissue archive, for North Atlantic right whales over the past 40 years. For this study, we selected 105 North Atlantic right whale females over 10 years of age with known calving histories and for whom we had tissue or DNA archived. Briefly, skin samples have been collected since the late 1980s via biopsy using specially designed biopsy tips attached to crossbow bolts (see [45] for more details). Sample collection is carried out in conjunction with photo-identification, to ensure the identity of the sampled whale is known. Samples are stored at Saint Mary's University (Halifax, Nova Scotia, Canada) in a 20% DMSO solution with 0.5M EDTA and saturated with NaCl [46]. If needed, DNA was extracted from skin biopsy samples in the same manner as the archived samples using standard phenol : chloroform methods (see [47] for more details). We included eight duplicate samples in our library to verify the consistency of our genotyping methods. These duplicates represent DNA from the same extraction, run through the library preparation steps in parallel.

### 2.2. ddRAD library preparation

We prepared our libraries for double digest restriction site associated DNA sequencing (ddRADseq) [48]. For each sample, 400ng of DNA was digested with NlaIII and EcoRI-HF (New England Biolabs, NEB). Double-stranded adapters were annealed to the cut sites with T4 DNA ligase (NEB). We cleaned each reaction with Ampure XP beads prior to attaching a unique combination of Nextera xt indexes (Illumina) to each sample and performed another bead clean up. We pooled pairs of samples and ran each pool in a separate lane of a Pippin Prep (2% agarose cassettes with ethidium bromide) selecting fragments 440–540 bp in size. Eluted product from two lanes of the Pippin Prep was pooled for a final bead clean-up. Four pools (each representing eight individually barcoded samples) were combined prior to normalizing. Libraries were sent to the McGill Genome Centre (Montreal, Canada) for normalizing and sequencing on one lane of a NovaSeq 6000 s4 150 × 150 bp run. The final library included 15% PhiX to increase library diversity. The supplementary information includes more details on library preparation (including electronic supplementary material, figure S1).

### 2.3. Read mapping and variant calling

Additional details, including schematics of the bioinformatics pipelines from raw reads through variant calling and filtering, are provided in the supplemental methods and in electronic supplementary material, figures S2 and S3.

Briefly, a near chromosome length assembly of the North Atlantic Right Whale genome is available from DNAZoo (DNAZoo.org). We used the ShortRead package [49] in R v3.6.0 to extract the reference sequences for the 21 longest scaffolds (2n = 42 in North Atlantic right whales; [50]). These 21 scaffolds account for approximately 91.4% of the entire assembly.

We used Trimmomatic v0.39 [51] on demultiplexed sequence reads to remove Illumina adapters, drop leading bases with base quality scores less than 20, drop reads with an average quality score less than 30 and to trim reads when the mean base quality in 5bp sliding window fell below 20. We mapped reads from all samples using bwa-mem and used SAMtools [52] to generate indexed bam files.

### 2.4. Variant calling

We wanted to compare the performance of two prominent variant calling approaches, and therefore performed variant calling using Stacks v2.64 [53] and Freebayes [54] both with a reference genome. Using both algorithms we also called invariant sites to better quantify the coverage of the genome. In the Stacks pipeline, we ran *gstacks* twice with different stringency thresholds setting both variant (--var-alpha) and genotype discovery (--gt-alpha) to 0.01 or 0.001. We ran the *populations* module for each *gstacks* data set requiring a locus to be present in 80% of individuals (-r 0.8) and generated a vcf file of all sites called (variant and invariant). In Freebayes, we called variants, including monomorphic sites, in consecutive 6 Mbp regions of the genome. In complex regions, we included additional constraints with the --use-best-n-alleles 4 flag and in some cases the --skip-coverage 10 000 flag to allow Freebayes to complete variant calling.

We undertook a series of filtering steps on each dataset outlined in electronic supplementary material, figure S3 resulting in four different datasets representing two different stringency thresholds produced by each of the calling algorithms. Briefly, all datasets were filtered for missingness, repetitive regions were removed based on the RepeatMasker files that accompanied the reference genome on DNAZoo, the datasets were limited to biallelic SNPs and filtered to only retain sites with a minor allele frequency greater than 0.01 and a minor allele count of at least 3. The Freebayes datasets were also filtered on mapping quality and depth (producing two Freebayes datasets based on different minimum genotype depth thresholds: DP5 and DP10). Stacks provides fewer annotations on the produced VCF files limiting the types of filters that can be applied, especially to invariant sites, and therefore instead of filtering on depth, we used lower alpha thresholds to increase the evidence needed to call a site or genotype as suggested by Rivera-Colón and Catchen [55].

Prior to filtering to biallelic SNPs, Stacks called nearly twice as many sites as Freebayes and nearly all of the sites called by Freebayes were also called by Stacks. In the final datasets, the Stacks pipelines resulted in nearly 50% more biallelic SNPs called than the datasets generated by the Freebayes pipeline (electronic supplementary material, table S4). We compared the genotype calls between two of the datasets: Freebayes DP10 and Stacks 0.001 representing the strictest filtering regimes for each calling algorithm and of the sites that were identified as variant sites by both methods, genotypes across individuals were called with high concordance having only 0.10–0.34% discordant genotypes (i.e. 7–27 discordant genotypes/4834–8089 assessed). True discordance rates are likely slightly higher however, as sites called as a variant by one pipeline and invariant by another were not compared.

We proceed with presenting results for Stacks called with the stricter alpha threshold (0.001) to retain a balance for strict filtering as well as retaining a larger number of SNPs. We present the main results from the other datasets in the electronic supplementary material, information.

Our sample set included duplicate samples which we can use to assess the consistency of our variant calling pipelines. We used BCFtools gtcheck [56] to compare genotypes of each pair of duplicates.

### 2.5. Calculating reproductive fitness

Following the de-lifing method put forth by Coulson *et al*. [43], we calculated the fecundity contribution of individual female right whales to overall population fecundity. Long-term studies on North Atlantic right whales, conducted by a range of entities, but with data organization and management being led by the New England Aquarium and the North Atlantic Right Whale Consortium (www.narwc.org), has amassed decades of sightings and calving data, providing detailed life-history information for individual whales. We limit our fecundity measures to between 1990, when field work became relatively consistent [27,37], and 2020, when survey effort was high, and sighting records in the database were considered complete. Sighting reports and field data take time to compile and verify, therefore database records for more recent years are not considered comprehensive.

Females were considered an adult at nine years of age if their birth year was known or eight years after initially being sighted if their birth year was unknown. A female was also considered an adult the year in which she had her first calf if this occurred before the nine-year threshold.

Population fecundity for each year was calculated as the number of calves that were observed divided by the number of adult estimated to be females alive in that year based on updates to the Pace *et al*. [27] model (R.M. Pace III *pers. comm.*). For example, given the optimal three-year reproductive cycle [31], in a ‘perfect year’ one third of all adult females would calve and the population fecundity would be calculated as 0.33.

We calculated the individual fecundity contribution for each of the 105 unique females in our genetic dataset as the relative contribution of a female to the population fecundity in a given year. In this way, a birth event is weighted more heavily in a year when few calves are born and given less weight in a year with a greater number of calving events. As the optimal reproductive cycle of a right whale female would be three years, we did not want to penalize a female for not having a calf in a year where many other calves were born because she was in a recovery or pregnancy year; therefore, we considered whether or not a female gave birth in a year with a sliding window approach meaning that her calving contribution for year_i_ would consider whether or not she had a calf in year_i−1_, year_i_ or year_i+1_. We calculated the mean individual fecundity contributions for each female over the years she was alive and adult (Equation 2.1), and omitted two females for which we had fewer than six years of fecundity values.

2.1 $$\mathrm{mean}\left(\frac{{\mathrm{reproduced}}_{\mathrm{year}(i-1|i|i+1)}(1\;|\;0)-{\mathrm{popfecundity}}_{\mathrm{year}}}{{\mathrm{NumAdultFemales}}_{\mathrm{year}i}-1}\right).$$

Equation (2.1) shows the mean annual fecundity contributions calculated for each female.

### 2.6. Calculating inbreeding coefficients

Individual inbreeding coefficients can be calculated with a number of different approaches. We used five different methods to calculate individual inbreeding coefficients for each female North Atlantic right whale in our genetic dataset to assess potential nuances in the way inbreeding may be presenting itself in the species. First, we calculated the inbreeding coefficient F for each individual using the --het flag in VCFtools v0.1.16 [57] to estimate the deviation between observed and expected heterozygosity within an individual. We used the *genhet* package [58] in R v4.2.2 [59] to calculate internal relatedness (IR: [60]) which incorporates allele frequency into its calculations and homozygosity by locus (HL: [18]) which also uses allele frequency by weighting the contribution of each locus to overall homozygosity. We used the package *InbreedR* [61] in R v3.6.0 to calculate standardized multi-locus heterozygosity (sMLH: [17,62]) which assesses relative heterozygosity across the genome of individuals. Finally, we used the package RZooRoH to identify the lengths of HBD segments using K = 11 (representing 10 HBD classes, and 1 non-HBD class). We calculated the proportion of the genome found in HBD tracts (F_HBD_) as the total length of HBD segments longer than 100 Kbp as a fraction of the total length of the 21-scaffold reference.

### 2.7. Estimating effect of inbreeding on fitness

We assessed the relationship between each inbreeding coefficient (F, sMLH, IR, HL and F_HBD_), and fecundity with a Bayesian regression model (equations (2.2) and (2.3)). As continuous variables, both individual fecundity and inbreeding coefficients were standardized (z-transformation) prior to being included in the model. The model was run in R v4.3.0 with the RStan package [63], with 2000 warm-up steps and 10 000 subsequent iterations.

2.2 mu=β0+(β1∗InbreedingCoefficient)

and

2.3 Individual Fecundity ∼ normal(mu,sigma).

Equations (2.2) and (2.3) show Bayesian models to estimate the effect of inbreeding coefficient on individual fecundity.

We gathered the posteriors of each model and calculated the mean of the posterior distribution and the 95% highest density interval (HDI) for the slope associated with each inbreeding coefficient and each dataset. To test whether specific genetic regions may be having a greater affect on fecundity, we conducted an association test using PLINK v1.9 [64] to look for correlations between genotypes at given sites and individual fecundity. We identified the genes associated with the most significant SNPs by intersecting them with the genome feature file that accompanied the reference assembly on DNAZoo, looking for putative genes located 100 Kb up- or down-stream from the positions of interest. We identified the potential function of these genes using the NCBI Gene database [65].

## 3. Results

After sequencing, we obtained a mean of 19.5M paired-end reads per sample (NCBI BioProject: PRJNA1027072), with nearly 90% of reads passing filters and a mean mapping quality of 54.69 after mapping to 21 scaffolds of the North Atlantic right whale reference assembly (table 1). For each variant calling program (Freebayes and Stacks), we produced two datasets with the stricter thresholds from each program (Freebayes DP10 and Stacks 0.001) yielding fewer SNPs after filtering, as expected (electronic supplementary material, table S4). The number of sites (both variant and invariant) called after removing repetitive regions was between 34.3 and 56.5 Mbp depending on the dataset, indicating that our results are based on approximately 1.59–2.61% of the 21-scaffold reference assembly. In order to balance a trade off between including a greater number of SNPs and individuals, as well as maintaining high confidence in variant calls, we opted to proceed with presenting results for the Stacks 0.001 dataset which called 26 011 biallelic SNPs across 93 individuals. The main results for the other datasets are presented in the supplementary information.

**Table 1.** Sequencing summary statistics.

number of samples sequenced	113
raw paired-end reads per sample (mean ± SD)	19 565 803 ± 10 884 334
percent of reads passing filters (mean ± SD)	89.87 ± 3.92
length of 21 scaffold assembly (bp)	2 166 782
mean mapping quality	54.69 ± 0.89

### 3.1. Fitness

**Figure 1.**
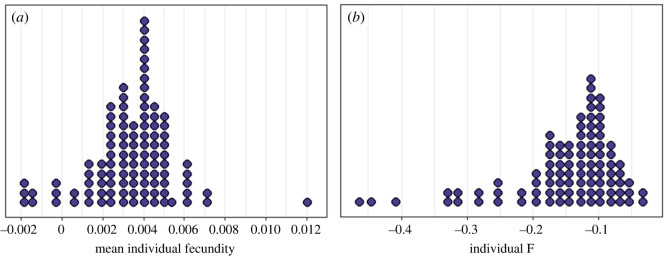
The distribution of (*a*) individual mean fecundity scores representing reproductive fitness for individual females (*n* = 103) and (*b*) individual inbreeding coefficient (F) calculated for each individual female (*n* = 93).

Individual fecundity estimates were able to be calculated for 103 females (figure 1*a*). There was clear variation in reproductive performance with individual fecundity values being shaped like a normal distribution across all samples.

### 3.2. Inbreeding coefficients

Each inbreeding coefficient showed quite a bit of variation across samples. *F* values for all individuals were negative (mean ± SD = −0.148 ± 0.083) indicating individuals were all more heterozygous than expected (figure 1*b*). All individuals also had negative IR values (mean ± SD = −0.151 ± 0.064) indicative of outbreeding or disassortative mating. The proportion of an individual's genome found in HBD segments (F_HBD>100Kb_) ranged from 0.06% to over 10% (mean ± SD = 0.027 ± 0.020). sMLH ranged from 0.910–1.258 (mean ± SD = 1.008 ± 0.068) and HL ranged from 0.466–0.621 (mean ± SD = 0.577 ± 0.030).

### 3.3. Inbreeding and fitness

**Figure 2.**
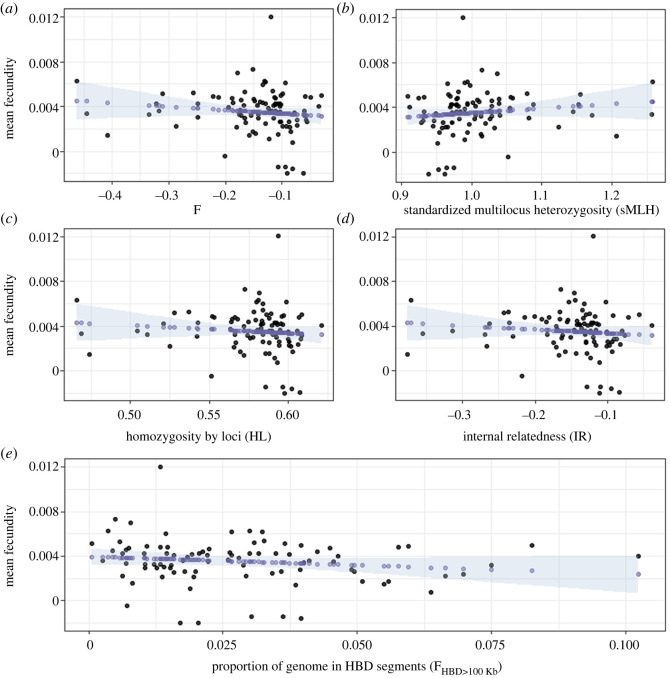
Relationship between individual fecundity and inbreeding coefficients (a. F, b. sMLH, c. HL, d. IR, e. F_HBD>100Kb_) using the Stacks 0.001 dataset. The posterior predicted values are indicated by the blue points and the 95% HDI is represented by the shaded area. Note that sMLH is a measure of heterozygosity, whereas the others are measures of homozygosity. So even though the slopes go in the opposite direction, they mean the same thing: as homozygosity increases, fecundity slightly decreases.

**Figure 3.**
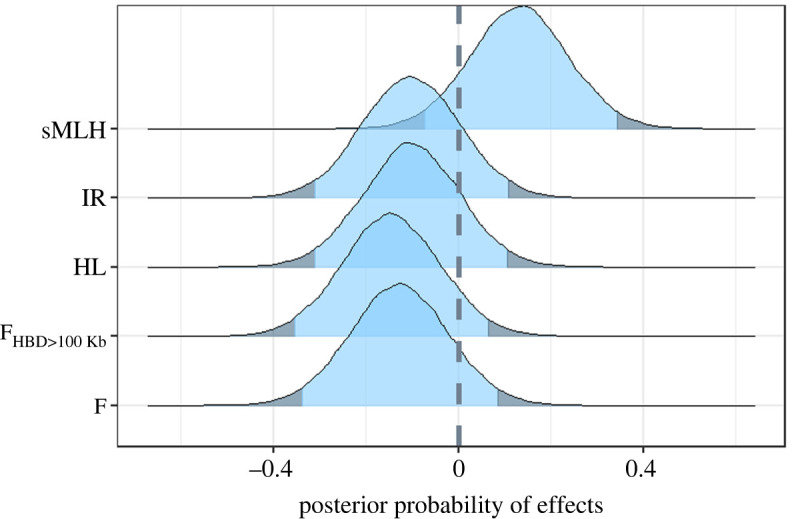
The effect of each inbreeding coefficient on individual mean fecundity for the Stacks 0.001 dataset. Tails of the posterior distributions falling in the highest or lowest 2.5% are shaded. Note that sMLH is a measure of heterozygosity, whereas the others are measures of homozygosity. So even though the effects go in the opposite direction, the magnitude of the effect size is very similar across all inbreeding coefficients.

The relationship between all inbreeding coefficients and fitness was very weak. F, HL, IR and F_HBD>100Kb_ all had a very slight negative relationship with individual fecundity, and the opposite pattern was exhibited for sMLH, suggesting that as inbreeding levels increase, an individual's fecundity only slightly decreases (figure 2). These trends were echoed by the posteriors of the Bayesian models, which all showed a peak probability of a negative effect, but for which the 95% HDI overlapped a slope of zero (figure 3). The results from all four datasets are presented in the supplemental information (electronic supplementary material, figures S4–S9). There are slight differences in the patterns found between each dataset, suggesting the specific sites included may be important for identifying inbreeding depression—therefore the effects of inbreeding on individual fecundity, while small, may be driven by effects at specific loci, rather than global patterns.

**Figure 4.**
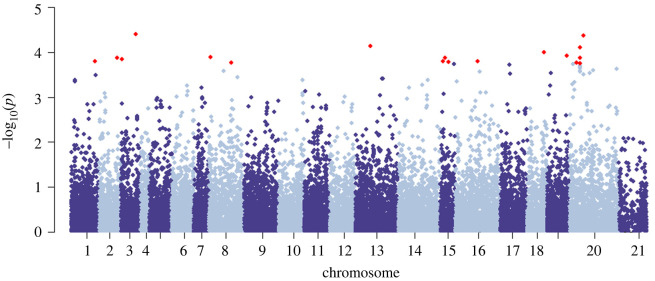
Association between genotype and reproductive fecundity across the genome. The 20 most significant SNPs are coloured in red and listed in table 2.

Our genome-wide association test revealed a number of SNPs with genotypes that were highly correlated with individual fecundity (figure 4). Table 2 lists the 20 SNPs with the lowest *p*-values and the putative genes identified by the genome feature file accompanying the reference assembly within ±100 Kb. Interestingly, seven of the most significant SNP associations were found on HiC_scaffold_20 and a few have putative involvement in reproduction: C2ORF80 may be associated with gonad development, PRMT7 may be involved in genomic imprinting and INO80E may be involved in DNA recombination [65]. The nature of ddRADseq means that coverage across the genome is not complete and therefore these sites identify possible genomic regions where haplotypes of specific genes may be having a direct effect on fitness.

**Table 2.** List of the top 20 SNPs with the lowest *p*-values identified in our GWAS and the gene annotations that were identified within ± 100 Kb per the annotations associated with the reference assembly. Genes listed in bold have putative associations with reproduction as described in the footnotes.

site	similar Genes within 100 Kb	*p* value
HiC_scaffold_20 : 39101094	—	1.75 × 10^−04^
HiC_scaffold_8 : 83910123	CBLB	1.72 × 10^−04^
HiC_scaffold_20 : 26579680	PLEKHM3, IDH1, C2ORF80^a^, CRYGA, PIKFYVE, Protein of unknown function	1.72 × 10^−04^
HiC_scaffold_15 : 29455338	AKTIP, RBL2, CHD9	1.68 × 10^−04^
HiC_scaffold_1 : 88360774	—	1.60 × 10^−04^
HiC_scaffold_15 : 9924377	NUDT7, VAT1L	1.58 × 10^−04^
HiC_scaffold_16 : 83116836	SMARCD2, S1PR1, CTNNAL1	1.57 × 10^−04^
HiC_scaffold_3 : 2991346	—	1.44 × 10^−04^
HiC_scaffold_15 : 17526456	SMPD3, SLC7A6OS, PRMT7^b^, ESRP2, SLC7A6, PLA2G15, Protein of unknown function	1.34 × 10^−04^
HiC_scaffold_20 : 39597891	—	1.33 × 10^−04^
HiC_scaffold_20 : 39597903	—	1.33 × 10^−04^
HiC_scaffold_2 : 67039621	HIRIP3, KCTD13, SEZ6L2, CDIPT, INO80E^c^, TAOK2, TMEM219, ASPHD1, T-ENOL, RAB2A, MVP, PRRT2, MAZ, KIF22, C16ORF54, COX5A, Protein of unknown function (x4)	1.33 × 10^−04^
HiC_scaffold_8 : 5989881	—	1.30 × 10^−04^
HiC_scaffold_19 : 72789327	FAM49A	1.20 × 10^−04^
HiC_scaffold_18 : 62196626	PREX2	1.02 × 10^−04^
HiC_scaffold_20 : 39573960	—	7.71 × 10^−05^
HiC_scaffold_20 : 39573966	-	7.71 × 10^−05^
HiC_scaffold_13 : 56211802	—	7.36 × 10^−05^
HiC_scaffold_20 : 50834179	TTN, Protein of unknown function	4.33 × 10^−05^
HiC_scaffold_3 : 53787103	ZC3H13, CPB2, LCP1	3.95 × 10^−05^

^a^associated with gonad development.

^b^involved in genomic imprinting.

^c^involved in DNA recombination.

## 4. Discussion

We identified a small, negative correlation between inbreeding coefficients and individual fecundity in female North Atlantic right whales, suggesting that although inbreeding may have a slight impact on female reproductive success, it is likely not the primary driver of reproductive variance found across individuals. This result was surprising given the extreme low levels of genetic diversity found in this species, its history of long-term small population size, and reproductive histories that varied highly across females, but which showed much smaller variation within each female (suggesting an intrinsic trait was likely influencing reproductive success). These patterns were consistently identified across a suite of inbreeding coefficients, and across datasets generated with different variant calling pipelines.

Reduced representation sequencing methods have become a cost-effective way of greatly increasing the number of markers able to be sequenced across a large number of samples where whole genome sequencing is still not feasible [66]. The efficacy, accuracy, and efficiency of different variant calling pipelines employed for RADseq data have often been compared (e.g. [53,67–71]) and with no clear winner emerging, the best pipeline for each dataset may ultimately depend on the expectations of the allele frequencies, sample size, coverage, availability of a reference genome, and likely many other factors. In this study, variant calling with Stacks2 [53] and Freebayes [54] yielded a different number of SNPs, and while we found high concordance in genotypes at variant sites, it is likely that rare alleles were handled differently by the two approaches and were therefore called differently by the two algorithms (e.g. some variable sites that were retained by Stacks2, may have been called by Freebayes as homozygous across all individuals). Casanova *et al*. [71] compared outputs produced by different variant calling pipelines for five different RADseq datasets, and as in our study, the different programs resulted in different SNP panels, but had little downstream effects on estimates of genetic differentiation. Conversely, Shafer *et al*. [69] suggest variant calling pipeline and downstream filtering can greatly influence the final outcomes from a study. Our approach of using two different pipelines, both detecting very minimal influence of inbreeding on fecundity, should lend further support to our findings.

This study suggests that adult female North Atlantic right whales are largely avoiding the negative consequences often associated with inbreeding. This could be a result of genetic purging whereby deleterious mutations have been selectively removed from the species' gene pool, but confirmation of this will require subsequent dedicated studies investigating genetic load. The inbreeding coefficients estimated for these females are also informative on their own with respect to understanding population dynamics within the species. For the past several decades, there have been fewer than 500 North Atlantic right whales [21,27], clearly limiting the opportunities for mates. While female reproduction may not be experiencing the effects of inbreeding depression, the viability of inbred calves may be affected. Observed heterozygosity was much greater than expected heterozygosity for all individuals in our dataset. This reinforces previous work based on microsatellites that right whales were more heterozygous than would be expected given the genotypes of their parents [72]. If female heterozygosity is not impacting her own reproductive fitness, then genotypes of the calves are likely dictating their survival. Frasier *et al.* [72] suggested post-copulatory selection for dissimilar gametes may be driving this pattern, however these findings may also be explained by fetal mortality being biased to fetuses with high inbreeding coefficients. Inbreeding depression acting on fetal survival or viability could help explain the decrease in successful calving events and the higher than expected heterozygosity rates seen in all of our samples. Excess heterozygosity was recently identified in an inbred population of Soay sheep (*Ovis aries*; [73]). As in this study, Stoeffl *et al*. [73] suggest this is likely due to increased embryonic or fetal mortality of inbred individuals. Future analyses comparing genome-wide data from known mother-father-calf triads would be able to estimate the degree to which inbreeding is impacting fetal survival, and therefore better quantify the impact of inbreeding on reproductive success (or reproductive failure), and better identify the specific regions of the genome involved (*sensu* [74–76]).

Excess genome-wide heterozygosity can be an artefact of sampling in small populations following a genetic bottleneck [77–80]. In this study, while we did only sample females which can increase this bias, these females represent overlapping generations and therefore are better representative of the allele frequencies in the entire population and not just in a single sex. Furthermore, in models that demonstrate excess heterozygosity, the modelled F statistics are much smaller than those we identified in this study—even for much lower effective population sizes [77,80]. For both of these reasons, we believe that while the small population of size of North Atlantic right whales could be contributing to the excess heterozygosity, the magnitude of this effect is likely driven by a loss of inbred fetuses.

It may also be possible that inbreeding depression on female reproductive fecundity is occurring, but due to effects at particular loci rather than at a genome-wide scale. Our GWAS suggests that there are SNPs throughout the genome where genotypes are highly associated with fecundity—especially on HiC_scaffold_20. Three of these sites were in close proximity to genes with potential involvement in reproduction (including C2ORF80). If mutations at C2ORF80 (also known as GONDA1 - gonad development associated 1) affect gonad development [65], this could in turn, interfere with a female's reproductive potential, and while we don't have direct evidence for this involvement, it could help explain the presence of nulliparous females. Here genotypes at a few SNPs near genes involved in reproduction and at a handful of sites on HiC_scaffold_20 had significant association with female fecundity, and therefore the potential involvement of genes from these regions (including those not captured by our RADseq panel) should be investigated further for heterozygosity fitness correlations to better understand the impact of inbreeding on reproductive fecundity.

Our results suggest that inbreeding coefficients may only explain a small part of a female's reproductive fecundity, but the question remains as to what is driving variance in reproductive success? A recent study suggests variation in body size may play a role in reproductive success [81]. North Atlantic right whales are heavily exposed to anthropogenic stressors, but this is variable across females. Over 80% of individual right whales have been entangled at least once in their lifetime [82] and all whales are likely exposed to vessel disturbance (in various ways and to varying degrees), throughout their annual migratory routes, critical habitat areas and their lifetime. These sublethal stressors affect all right whales and can influence their fecundity through acute interactions [26], or through lifetime cumulative stress leaving epigenetic or other signatures of stress which could in turn affect fecundity. Models are being developed to quantify individual fitness and recovery throughout the lifetime of individual right whales incorporating visual health assessments (e.g. [26,83,84]). While inbreeding is having only a very small effect on reproductive fecundity, it could be affecting other fitness traits, or could be used as a correction factor for future individual based models.

This study is part of a larger effort to understand genetic limitations to population recovery in North Atlantic right whales and the scope of this study was to use genome-wide markers to investigate the effects of inbreeding on reproduction. We found that inbreeding is only having a slight impact on the reproductive performance of female North Atlantic right whales. This is good news for the conservation and the long-term viability of the species, suggesting that the variation in female reproductive success is likely due to anthropogenic or other external factors such as food availability, many of which, unlike inbreeding, can be addressed through protection measures. However, our genomic data support results from earlier analyses suggesting that fetal mortality is biased towards inbred individuals, and therefore that inbreeding is impacting reproduction, just in a different manner than explicitly examined here. This finding is a double-edged sword, where such a pattern would have a negative short-term effect of lowering reproductive performance of the species (due to fetal loss), but a positive long-term effect (observed here) of maintaining heterozygosity at much higher levels than would be expected for a species with a small effective population size and this demographic history. Genomic analyses focused on addressing this pattern in more detail should be a high priority, as should teasing apart the relationship between stressors and female fecundity.

## Data Availability

Raw sequence data have been archived in NCBI's Sequence Read Archive as BioProject PRJNA1027072. Data and relevant code for this research work are stored in GitHub: https://github.com/carlacrossman/NARW_ddRAD_InbreedingDepression and have been archived within the Zenodo repository: https://doi.org/10.5281/zenodo.11263629 [85]. The data are provided in electronic supplementary material [86].
